# EHMTI-0189. Tryptophan-kynurenine metabolism in migraine patients

**DOI:** 10.1186/1129-2377-15-S1-E17

**Published:** 2014-09-18

**Authors:** Z Majláth, G Veres, D Zádori, A Balog, N Watti, J Tajti, L Vécsei

**Affiliations:** 1 Department of Neurology, University of Szeged, Szeged, Hungary; 2 Faculty of Medicine, University of Szeged, Szeged, Hungary; 3 Neuroscience Research Group of the Hungarian Academy of Sciences (MTA – SZTE Neuroscience Research Group), University of Szeged, Szeged, Hungary

## Introduction

Alterations of the serotoninergic system are well-known in migraine, however, the other main route of tryptophan metabolism, the kynurenine pathway has not yet been investigated in migraine. Psychiatric comorbidity, especially depression is highly frequent in migraine patients, but the exact pathomechanism is not fully clarified.

## Aims

To investigate kynurenine metabolites in migraine patients, and to assess psychiatric comorbidity.

## Methods

The study was approved by the Ethical Committee of the University of Szeged. 47 migraine patients and 43 healthy controls have been involved, written informed consent was obtained from every participant. Headache patients were classified according the IHS criteria. Depression, anxiety, stress, and quality of life were assessed by validated questionnaires: the Beck Depression Inventory, the Holmes and Rahe Stress Scale, the SF-36, and the Spielberger State-Trait Anxiety Inventory. Serum samples have been analyzed by HPLC.

## Results

31% of migraine patients presented depressive symptoms. Migraine patients had a significantly lower quality of life, which showed a strong correlation with psychiatric comorbidity. Tryptophan levels were significantly lower in migraineurs than in controls. Serum indoleamine 2,3-dioxygenase (IDO) activity, calculated by kynurenine/tryptophan ratio, was significantly higher in migraine patients with depressive symptoms, while in non-depressed patients IDO activity was not statistically different.

## Conclusions

Psychiatric comorbidity, especially depression is highly prevalent among migraine patients, and contributes to the worsening of quality of life. The alterations in tryptophan-kynurenine metabolism might contribute to pathomechanism of migraine and depression comorbidity.

No conflict of interest.

